# Structural Characterization and Efficacy in Alleviating Lung Inflammation of Sialylated Glycopeptides from Edible Bird’s Nest

**DOI:** 10.3390/nu17101745

**Published:** 2025-05-21

**Authors:** Qiushi Li, Chenxi Zhang, Guandong Fang, Shuang Qiu, Man Yuan, Nan Qian, Dongliang Wang, Xiangrong Cheng

**Affiliations:** 1 School of Food Science and Technology, Jiangnan University, Wuxi 214122, China; liqiushi1023@163.com (Q.L.); chenxi.zhang@chinyou.com (C.Z.); fgd12232023@163.com (G.F.); qiannan202412@163.com (N.Q.); 2 State Key Laboratory of Food Science and Resources, Jiangnan University, Wuxi 214122, China; 3 Hebei Edible Bird’s Nest Fresh Stew Technology Innovation Center, Langfang 065700, China; qiushuang0614@163.com (S.Q.); codeyduck@163.com (M.Y.)

**Keywords:** edible bird’s nest, sialylated glycopeptide, lung inflammation, metabolomics

## Abstract

**Objectives:** This study aimed to characterize the basic structure of sialylated glycopeptide (SCP) from edible bird’s nest, and to explore the intervention effect and mechanism of SCP based on a mouse lung inflammation model induced by lipopolysaccharide (LPS). **Methods:** C57BL/6 mice were randomly divided into the control group (CON), model group (LPS), EBN group, SCP group, and SA group. **Results:** The results showed that SCP had the typical structures of polypeptides and carbohydrates. SCP effectively intervened in the lung inflammation response. The number of neutrophils (Neu) in BALF decreased by 41.3%, the level of tumor necrosis factor-α (TNF-α) decreased by 36.4%, and the W/D ratio of lung tissues decreased by 27.2%, effectively preventing pathological changes in lung tissues. A total of 40 differential metabolites such as choline, linolenic acid, and xanthine were screened between the SCP group and the LPS group. These differential metabolites were mainly enriched in the metabolic pathways of glycerophospholipids, alpha-linolenic acid, and purines. **Conclusions:** The research results support that SCP, as the active substance of edible bird’s nest, can effectively improve lung inflammation, providing theoretical guidance for the development of functional edible bird’s nest products.

## 1. Introduction

Edible bird’s nest (EBN) is coagulated from the saliva secreted by several species of swiftlets in the genus *Aerodramus* of the *Apodidae* family mixed with other substances. As a traditional precious product with both medicinal and edible properties, it has a long history of consumption in Asian regions, especially in China [1]. The main component of EBN is primarily composed of macromolecular glycoproteins rich in sialic acid (SA), which typically exist as terminal residues at the non-reducing ends of glycan chains in these glycoproteins [2]. The SA content in EBN, a pivotal component of its bioactive glycopeptides, exhibits notable variability influenced by species origin, geographical provenance, and nesting environment [3]. For example, *Aerodramus fuciphagus* nests contain 1.9-fold higher conjugated SA (dry weight) than *A. maximus* nests, reflecting genetic differences in salivary glycoprotein biosynthesis. House-farmed EBN from Malaysia exhibits 63% higher SA levels than cave-nested counterparts, a disparity linked to environmental factors like dietary composition and microhabitat [4]. Regionally, Vietnamese EBN harbors elevated sialoglycoprotein content (the primary molecular carriers of SA) compared to major producing regions such as Malaysia [5]. These variations in SA abundance and glycan architecture are critical determinants of glycopeptide structures, potentially influencing biological activities like inflammation modulation [6]. Compared with the untreated EBN, the enzymatically hydrolyzed EBN extract has higher biological activities such as anti-inflammatory, antioxidant, and immunological activities [7]. Glycopeptides prepared from the extraction of many natural foods, including *Lycium barbarum* and egg glycopeptides, have been demonstrated to play anti-inflammatory and immune-promoting roles [8,9]. Notably, EBN has the efficacy of moistening the lungs and nourishing yin in Chinese medicine. In existing studies, EBN can treat respiratory diseases by inhibiting inflammation, regulating the immune system, and maintaining oxidative balance [10,11]. Currently, research on the anti-inflammatory activities of EBN mainly focuses on peptides, but the role of glycopeptides has not been emphasized. Previous studies have proven that the sialylated glycopeptide (SCP) of the EBN is the main active component in edible bird’s nest, and it can exert a neuroprotective effect through the gut–brain axis [12]. However, currently, the efficacy of SCP in alleviating lung inflammation remains unclear and requires further research.

Lung inflammation is an important pathological basis for many severe respiratory diseases, which can significantly affect the metabolic homeostasis of the body and weaken the body’s functions. Its pathogenesis is extremely complex, involving multiple aspects including immune response, oxidative stress, cell apoptosis, and microbial infection [13]. When the lungs are invaded by pathogens or other harmful stimuli, the immune system is activated, triggering an inflammatory response, which subsequently leads to phenomena such as immune disorders, tissue damage, and metabolic abnormalities [14]. Currently, although traditional clinical intervention methods such as glucocorticoids and antibiotics can alleviate symptoms, long-term use is likely to trigger drug resistance and side effects. In recent years, extensive attention has been paid to searching for food-derived components with anti-inflammatory properties from the diet. A variety of food-derived glycoprotein complexes have been proven to possess anti-inflammatory effects [15]. Many natural foods, such as *Ganoderma lucidum*, contain abundant bioactive components and have been confirmed to be capable of intervening in lung inflammation [16]. Lipopolysaccharide (LPS), which serves as a key constituent of the cell wall in Gram-negative bacteria, can activate the immune system and trigger inflammatory reactions such as the massive release of pro-inflammatory cytokines and all kinds of tissue damage [17]. Thus, LPS is commonly used to induce lung injury [18,19].

Metabolomics has been extensively utilized to comprehensively investigate the patterns of metabolic dynamic responses in organisms when they are under exogenous stimuli or endogenous pathological changes [20]. It enables the precise identification and quantitative investigation of the metabolic components of small molecules in biological samples [21]. Subsequently, the matching of metabolic pathways and the elucidation of molecular mechanisms can be achieved. Existing studies have proven that when pulmonary inflammation exists in the organism, it will be accompanied by metabolic disorders. Therefore, investigating the metabolic changes in lung tissues helps understand the mechanism of lung inflammation induced by LPS and evaluate the regulatory effect of SCP.

In this study, the structure of SCP was characterized by combining the chemical composition and primary structure. A mouse model of lung inflammation was established using lipopolysaccharide. The intervention effect of SCP on lipopolysaccharide-induced lung inflammation in mice was evaluated from three aspects: the level of lung inflammation, the pathological structure of lung tissues, and the metabolic level of lung tissues.

## 2. Materials and Methods

### 2.1. Materials and Reagents

Edible bird’s nests horn (from Malaysia) was purchased from a local supermarket in Wuxi. Sialic acid was purchased from Kesitan Biotechnology Co., Ltd. (Wuhan, China), and Alcalase 2.4L (2.4 AU/g), Papain (800 U/mg) were purchased from Novozymes (Bagsværd, Denmark). Beyoblue™ coomassie blue super fast staining solution, prestained color protein molecular weight marker (10–170 kDa), SDS-PAGE protein loading buffer (2×), periodic acid-Schiff staining kit, and BCA protein assay kit, protein precast gel (4–20%, 15 Wells) were purchased from Beyotime Biotechnology Co., Ltd. (Shanghai, China). Ethanol, NaHSO_4_, o-phenylenediamine hydrochloride (OPA), glucose, phenol, sulfuric acid, etc., were all purchased from Sinopharm Chemical Reagent Co., Ltd. (Shanghai, China). Lipopolysaccharide (LPS) was purchased from Sigma-Aldrich (St. Louis, MO, USA). TNF-α, IL-1β, and IL-6 commercial kits were purchased from Xiamen Hui Jia Biotechnology Co., Ltd. (Xiamen, China). Acetone was procured from Zhejiang Hannuo Chemical Technology Co., Ltd. (Jinhua, China).

### 2.2. Experimental Animals

Forty 7-week-old male C57BL/6J mice (specific pathogen-free [SPF] grade) were obtained from Jiangsu GemPharmatech Co., Ltd. (Nanjing, China). The experimental procedures were approved by the Laboratory Animal Welfare and Ethics Committee of Jiangnan University (approval number: JN.No20230415c0800705[145]).

### 2.3. Preparation of Sialylated Glycopeptides (SCP) from EBN

The sequential enzymatic hydrolysis of EBN horn was performed using Alcalase 2.4 L followed by papain under optimized conditions. The first-step hydrolysis with Alcalase 2.4 L was conducted at 60 °C and pH 8.5 for 12 h with an enzyme–substrate ratio of 6000 U/g. Subsequently, papain hydrolysis was performed at 50 °C and pH 7.0 for 2 h using an increased enzyme-substrate ratio of 10,000 U/g. Enzyme inactivation was achieved through thermal denaturation (95 °C, 10 min). Finally, purification was achieved through our laboratory’s established method [12]. After being frozen at −80 °C for 12 h, the extracted SCP powder was obtained by a lyophilizer (SCIENTZ-10N, SCIENTZ Biotech Co., Ltd. (Ningbo, China)).

### 2.4. Structural Characterization of SCP

#### 2.4.1. Neutral Sugar and SA Content Quantification

The content of neutral sugars was measured via the phenol-sulfuric acid approach. To SCP samples, 1 mL of 5% phenol solution was added, followed by the rapid addition of 5 mL of concentrated sulfuric acid. After thorough mixing, the mixture was incubated in a water bath at 37 °C for 30 min. Subsequently, the absorbance was measured at 490 nm using a Microplate Reader 96 (EPOCH1, Bio Tek Instruments, Inc., Winooski, VT, USA). Glucose (anhydrous, dried to constant weight in an oven) was used as the standard reference [22].

SA content was determined by liquid chromatography (LC-20AT, Shimadzu, Kyoto, Japan). SCP sample (50 mg) was hydrolyzed with 5 mL 0.5 M NaHSO_4_ at 80 °C for 30 min, after which, derivatization was performed with 5 mL 10 g/L OPA (in 0.25 M NaHSO_4_) at 80 °C for 40 min. After filtration through the 0.22 μm filters, the solution was diluted to 25 mL and stored in the dark. In the HPLC analysis, the EF-C18Bio column (Galaksil, Rugao City, China) was applied at 30 °C. The mobile phase, acetonitrile–water (5:95, *v*/*v*), flowed at a 1.0 mL/min rate. Detection was performed at 230 nm with a 20 μL injection volume [12].

#### 2.4.2. Amino Acid Composition Analysis

Approximately 100 mg of solid powder was hydrolyzed with 8 mL 6 M HCl under N_2_ at 120 °C for 22–24 h. After neutralization with 10 M NaOH and dilution to 25 mL, the sample was filtered and centrifuged (10,000 rpm, 10 min). For tryptophan, 8 mL 5 M NaOH was used for initial hydrolysis, followed by neutralization with 6.7 mL 6 M NaOH. In the HPLC analysis (Agilent, Santa Clara, CA, USA), the ODS column (Agilent Hypersil, Santa Clara, CA, USA) was employed with gradient elution using mobile phases A (27.6 mmol/L sodium acetate-triethylamine-THF, 500:0.11:2.5, *v*/*v*/*v*, pH 7.2) and B (80.9 mmol/L sodium acetate-methanol-acetonitrile, 1:2:2, *v*/*v*/*v*, pH 7.2), 0–17 min (8–50% B), 17–20.1 min (50–100% B), 20.1–24 min (100–0% B). The flow rate was 1.0 mL/min at 40 °C, with UV detection at 338 nm. Quantification was via external calibration.

#### 2.4.3. Molecular Weight Distribution Determination

SCP sample was vortexed with loading buffer (1:1, *v*/*v*), heat-denatured (100 °C, 3 min) and then centrifuged (8000 rpm, 3 min) to pellet aggregates. The molecular weight of SCP was determined through a discontinuous SDS-PAGE system (5% stacking/10% resolving gel) under reducing conditions, with a sample loading volume of 10 μL. Coomassie Brilliant Blue was used to stain protein, and Periodic acid-Schiff (PAS) reagent was used for glycan [23].

#### 2.4.4. FTIR Spectral Analysis

ATR-FTIR spectroscopy (Thermo Fisher Scientific, Waltham, MA, USA) was employed to analyze secondary structures. Lyophilized SCP samples were directly placed on the ATR crystal and scanned over 4000–650 cm^−1^ with 32 scans at 4 cm^−1^ resolution [24].

### 2.5. Animal Grouping and Biological Sample Collection

**Figure 1:**
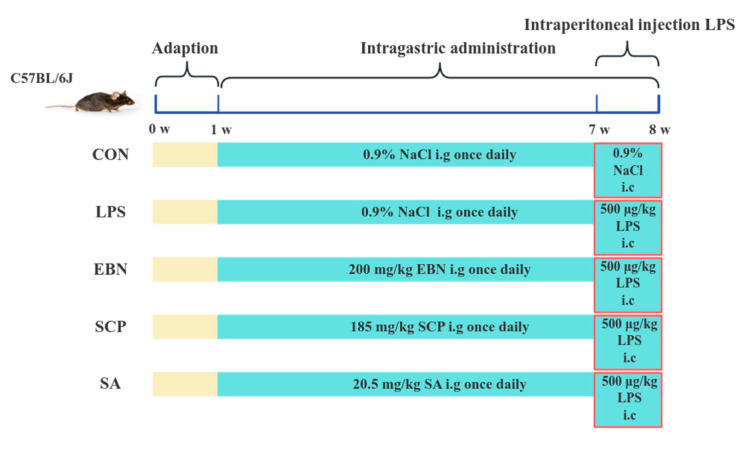
Animal grouping and intervention scheme.

Mice were kept at Jiangnan University’s Laboratory Animal Centre (SYXK-(Su)-2021-0056) in a barrier environment. They experienced a 12 h light/dark cycle. The temperature ranged from 20 to 26 °C, and humidity was 40–70% under the artificial 12 h light/dark regime. Forty mice were first individually tagged with unique ear tags (numbered 1–40), and a random sequence was derived from a random number table. Mice were then assigned to five experimental groups (*n* = 8) based on this sequence. Mice were acclimated for 1 week in a standard environment with free access to food and water before the experiment. During the experiment, stress responses were monitored by observing behavioral status, such as activity level, food intake, and body weight changes. Procedures were performed gently to avoid unnecessary stress. The control group (CON) and LPS model group (LPS) were given 0.2 mL of saline by oral gavage daily, while the EBN group (EBN) was administered 200 mg/kg/d EBN homogenate (dry weight basis), the SCP group (SCP) received glycopeptides equivalent to EBN’s sialic acid content, and the SA group was given an equimolar free sialic acid solution for 7 consecutive weeks. During the last week, all groups except CON were intraperitoneally injected with LPS (500 μg/kg/d) for 7 days. After the last LPS injection, the mice were fasted overnight. Following weighing, they were anesthetized by isoflurane inhalation. Subsequently, the mice were euthanized via cervical dislocation and immediately dissected. After sacrificing the mice, the bronchoalveolar lavage fluid (BALF) and lung tissue were collected (see Figure 1).

BALF collection: Mice were euthanized, and limbs were secured with pins. The chest cavity and tracheal skin were incised, and the right lung was gently ligated with silk thread. After exposing the trachea, blunt forceps were used to puncture it, followed by the insertion of a tracheal cannula. Once stabilized, 0.5 mL of PBS buffer was injected via a syringe, held for 15–30 s, and then aspirated into a 1.5 mL microcentrifuge tube. This procedure was repeated three times.

Lung tissue homogenization: Frozen lung tissue was thawed and homogenized in PBS buffer (1:100, *m*/*v*). After mincing, 2–3 zirconia beads were added, and the tissue was disrupted using a tissue homogenizer (Jingxin Industrial, Dongguan City, China) at 70 Hz for 90 s (2–3 cycles). Then, it was centrifuged (4 °C, 4000 rpm, 20 min), and the supernatant was collected for biochemical assays.

### 2.6. Lung Inflammatory Markers

BALF was used to conduct cell count using an automatic hematology analyzer (BC-5000 Vet, Mindray Bio-Medical Electronics Co., Ltd., Dongguan City, China), including white blood cells (WBC), monocytes (Mon), neutrophils (Neu), eosinophils (Eos), basophils (Bas), and lymphocytes (Lym). Lung tissues were used to determine TNF-α, IL-1β, and IL-6 levels by ELISA kits (Xiamen Huijia Biotech, Xiamen, China, OD450 nm).

### 2.7. Lung Histopathology

Lung tissue was rinsed with PBS buffer, followed by blotting to remove surface moisture. The wet weight of the tissue was then measured. Subsequently, the tissue was dried at 80 °C for 48 h until a constant weight was achieved (dry weight). The wet/dry weight (W/D) ratio of the lung tissue was calculated accordingly [25].

Fresh lung tissues were fixed by immersion in 4% paraformaldehyde for 24 h. After fixation, the tissues underwent gradient ethanol dehydration, xylene clearing, paraffin embedding, and tissue sectioning. Subsequently, the sections were deparaffinized, rehydrated, and stained with hematoxylin solution for 10 min. Differentiation was performed using 1% hydrochloric acid-alcohol for 30 s, followed by rinsing with tap water and bluing with ammonia water. After rinsing again, the sections were stained with eosin for 3 min. Following dehydration, clearing, and mounting, the pathological changes in lung tissues from each group were observed under an optical microscope.

### 2.8. Lung Metabolomics

Lung tissue metabolomic profiling was performed according to the established LC-MS/MS protocol [26]. Chromatographic raw data were processed through Compound Discoverer 3.3 (Thermo Fisher Scientific) for baseline correction, peak alignment, and feature extraction. Subsequent analytical workflows included quantitative analysis of peak areas coupled with molecular formula prediction, with spectral matching conducted against the mzCloud, mzVault, and MassList databases, supplemented by a proprietary compound library (Shanghai Senschip Biotech, Shanghai, China).

### 2.9. Statistical Analysis

Data were presented as the mean ± standard deviation (SD). For data that conformed to a normal distribution, statistical analyses were performed as follows: For datasets with three or more groups, one-way analysis of variance (ANOVA) was conducted, followed by post-hoc pairwise comparisons using the Least Significant Difference (LSD) test and Duncan’s multiple range test. For two-group comparisons, a *t*-test was applied. When the data did not meet the assumptions of parametric tests, the non-parametric Kruskal–Wallis (KW) rank sum test was utilized. A significance level of *p* < 0.05 was set to determine statistical differences among groups. Multivariate statistical analyses were carried out using MetaboAnalyst 6.0 and SIMCA 13.0 software.

## 3. Results

### 3.1. Chemical Composition Analysis of SCP

**Figure 2:**
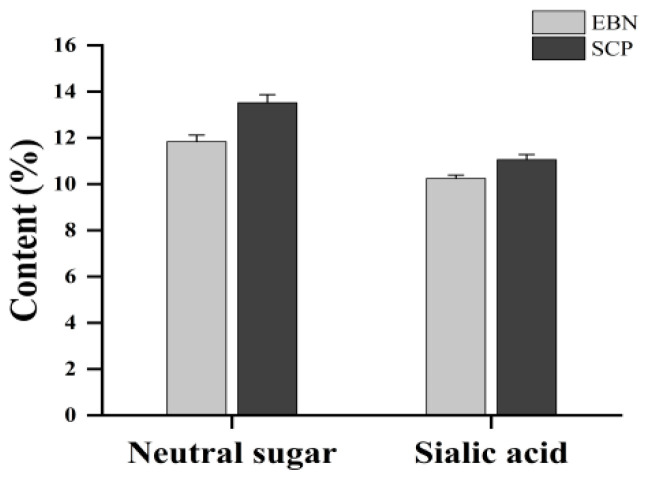
Neutral sugar contents and sialic acid contents of EBN and SCP.

The sialic acid (SA) content in EBN was determined to be 10.25 ± 0.14%, with neutral sugars accounting for 11.84 ± 0.28%. In contrast, an increase in SA content (11.06 ± 0.22%) and a concomitant rise in neutral sugar levels (13.52 ± 0.35%) were observed in SCP (Figure 2). These results indicated an enriched glycan proportion in SCP compared to native EBN.

An analysis of 18 amino acids in SCP was performed (Table 1). The top five amino acids by abundance in SCP were proline (Pro), aspartic acid (Asp) or asparagine (Asn), glutamic acid (Glu) or glutamine (Gln), valine (Val), and leucine (Leu). Additionally, SCP was rich in nine essential amino acids (with histidine (His) being essential for infants), which accounted for 42.22 ± 0.02% of the total amino acid content. The combined content of Asp or Asn, threonine (Thr), and serine (Ser) represented 23.34 ± 0.08% of the total amino acid content, suggesting the potential presence of glycosylation modifications in SCP.

### 3.2. Primary Structure Analysis of SCP

**Figure 3:**
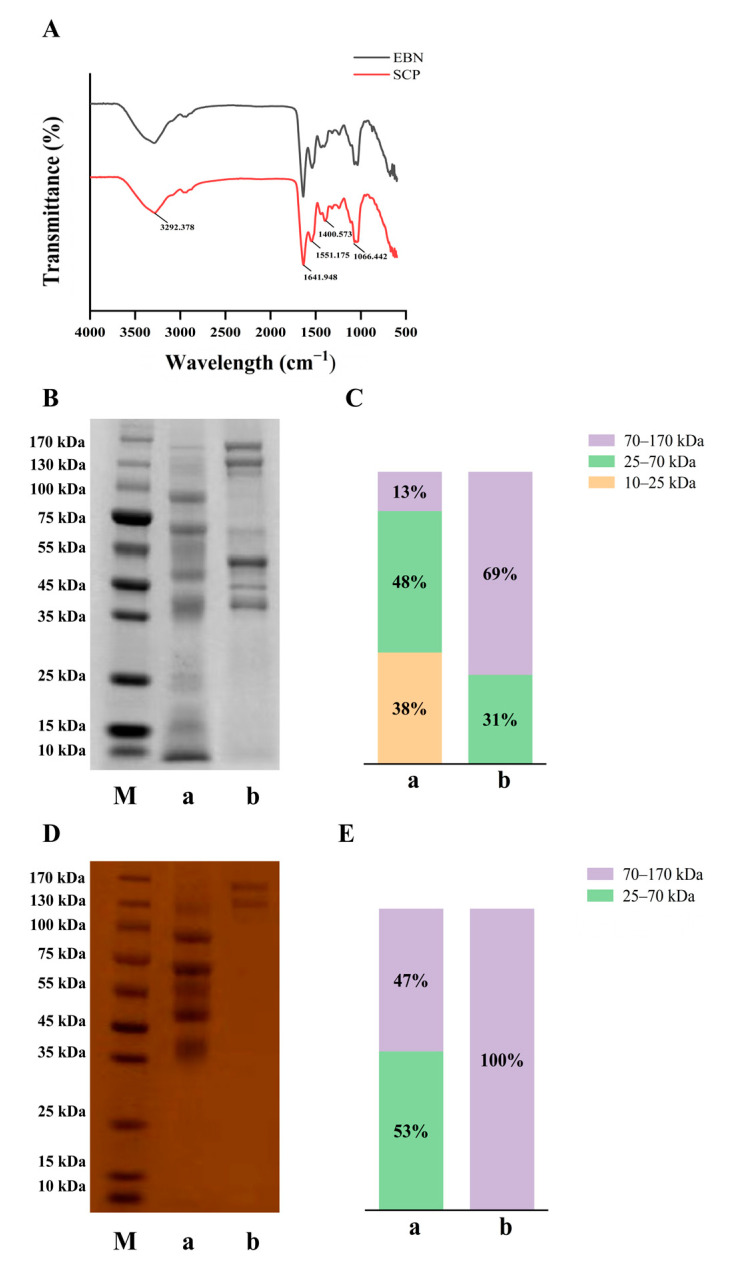
FT-IR spectral and molecular weight distribution of EBN and SCP. (**A**) The Fourier infrared spectra analysis of EBN and SCP. (**B**) Electrophoresis of EBN and SCP stained with carmine blue. (**C**) Relative molecular weight distribution of EBN and SCP under carmine blue staining. (**D**) Electrophoresis of EBN and SCP under PAS staining. (**E**) Relative molecular weight distribution of EBN and SCP under PAS staining. M: Marker, a: EBN, b: SCP.

FT-IR spectra of EBN and SCP revealed significant differences (Figure 3A). In EBN, a broad peak at 3600–3000 cm^−1^ was attributed to hydroxyl (−OH) stretching vibrations of polysaccharides [27]. Absorption bands at 1695–1630 cm^−1^ (amide I, C=O stretching) and 1560–1500 cm^−1^ (amide II, N-H bending) confirmed the presence of proteins/polypeptides [28,29]. For SCP, enhanced absorption bands at 1401 cm^−1^ (−COO− symmetric stretching), and 1066 cm^−1^ (C-O), coupled with a carbohydrate-specific peak at 1085–1050 cm^−1^, demonstrated its glycopeptide nature [29]. Vibrations in the 1440–1395 cm^−1^ region likely corresponded to carboxyl groups from sialic acid (SA) and alduronic acids, consistent with characteristic glycopeptide spectral profiles. These results further supported the glycopeptide structure of SCP.

The molecular weight profiles were analyzed by SDS-PAGE (Figure 3B,C). EBN proteins’ molecular weight was mainly between 70–170 kDa (69%). Following enzymatic hydrolysis, the proportion of this range was reduced to 13%, with a concurrent increase in the 25–70 kDa fraction to 48% and the emergence of low molecular weight fragments (10–25 kDa, 38%), confirming effective protein size reduction. PAS staining (Figure 3D,E) revealed that glycans uniformly occupied the 70–170 kDa range. Post-enzymatic treatment, this proportion decreased to 47%, while the 25–70 kDa fraction increased to 53%. Notably, glycans were not degraded below 25 kDa, suggesting covalent stabilization between glycan and protein backbones.

### 3.3. SCP Alleviated the Changes of LPS-Induced Lung Inflammation Levels

**Figure 4:**
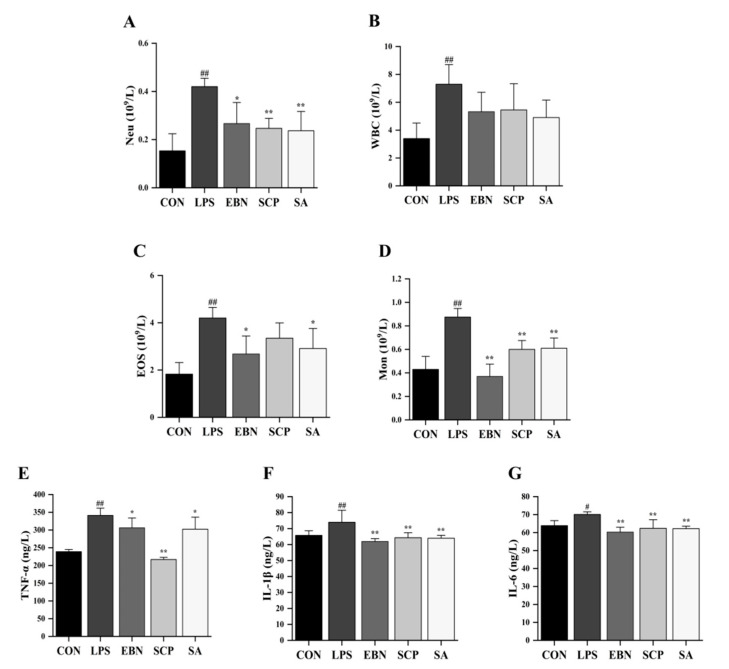
Changes in lung inflammation levels of mice in each group. (**A**–**D**) Counts of inflammatory cells (Neu, WBC, EOS, and Mon) in the BALF of mice in each group. (**E**–**G**) Levels of pro-inflammatory cytokines (TNF-α, IL-1β, and IL-6) in the lung tissue of mice in each group. *n* = 8; results are presented as mean ± SD. # *p* < 0.05, ## *p* < 0.01 vs. CON group, * *p* < 0.05, ** *p* < 0.01 vs. LPS group.

The counts of inflammatory cells in bronchoalveolar lavage fluid (BALF) among different experimental groups were determined (Figure 4A–D). LPS induction resulted in a statistically significant increase (*p* < 0.01) in the counts of neutrophils (Neu), white blood cells (WBC), eosinophils (Eos), and monocytes (Mon), suggesting that LPS successfully induced inflammatory cell disorder in BALF. Compared with the LPS group, intervention groups involving EBN, SCP, and SA were observed to significantly decrease the number of Mon in the BALF (*p* < 0.01). Furthermore, a significant reduction in the number of Neu within the BALF was also noted following interventions with EBN (*p* < 0.05), SCP (*p* < 0.01), and SA (*p* < 0.01). Specifically, under SCP intervention, the numbers of Neu in the BALF were reduced by 41.3%. It was indicated that EBN, SCP, and SA effectively modulated the aggregation of inflammatory cells within the BALF of mice induced by LPS. Among these interventions, SCP exhibited the most significant inhibitory effect on Neu recruitment.

Important pro-inflammatory cytokines in lung tissues of all groups, such as TNF-α, IL-1β, and IL-6, were determined (Figure 4E–G). Compared with the CON group, the levels of TNF-α (*p* < 0.01), IL-1β (*p* < 0.01), and IL-6 (*p* < 0.05) in the lung tissues of mice significantly increased after LPS induction. It was indicated that the intraperitoneal injection of LPS successfully induced inflammation in the lung tissues of mice. After the intervention of EBN, SCP, and SA, the levels of IL-1β and IL-6 in the lung tissues of mice were significantly decreased compared with those in the LPS group. Notably, compared with the LPS group, the level of TNF-α in the lung tissues decreased by 36.4% after the intervention of SCP, which was 3.6 times and 3.2 times the reduction ratios of EBN and SA intervention groups, respectively. It was suggested that the intervention groups of EBN, SCP, and SA all had a certain regulatory effect on the increase in the levels of pro-inflammatory cytokines. Among them, the intervention of SCP had the most significant effect on improving the level of TNF-α in the lung tissues.

### 3.4. SCP Alleviated the Changes of LPS-Induced Lung Tissue Pathological Structures

**Figure 5:**
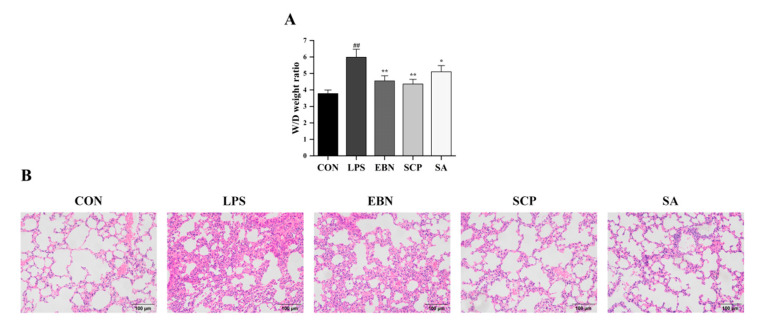
Changes in lung tissue pathological structures of mice in each group. (**A**) The ratio of W/D weight in the lung tissue of mice in each group. (**B**) H&E staining in the lung tissue of mice in each group (200×). *n* = 8; results are presented as mean ± SD. ## *p* < 0.01 vs. CON group, * *p* < 0.05, ** *p* < 0.01 vs. LPS group.

To evaluate the protective effect of SCP against lung tissue injury induced by LPS, the wet-to-dry (W/D) weight ratio of lung tissues was measured (Figure 5A). Compared with the CON group, a significant increase in the W/D ratio was detected in the LPS group (*p* < 0.01), suggesting that an edema phenomenon might occur in the lung tissue. All intervention groups (EBN, SCP, and SA) mitigated this increase (*p* < 0.05). Notably, the SCP group showed the most remarkable improvement, with a 27.2% decrease in the W/D weight ratio.

Hematoxylin and Eosin (H&E) staining of lung tissues in each group further revealed structural alterations of the lung tissues (Figure 5B). The CON group exhibited intact alveolar architecture, orderly arranged epithelial cells, and no edema [30]. In contrast, the LPS group displayed collapsed alveoli, thickened alveolar walls, and increased interstitial exudation. Intervention groups (EBN, SCP, and SA) showed varying degrees of mitigation in tissue damage. Notably, the SCP group demonstrated near-normal alveolar structure with reduced inflammatory cell infiltration. All changes in the pathological structure of the lung tissues revealed that the intraperitoneal injection of LPS successfully led to pathological changes in the structure of the lung tissues. Compared with the LPS group, the lung tissue structures of the intervention groups of EBN, SCP, and SA all showed certain improvements. In addition, the lung tissue structure of the SCP intervention group was the closest to that of the CON group.

### 3.5. SCP Alleviated the Change of LPS-Induced Lung Tissue Metabolic Levels

#### 3.5.1. Analysis of the Metabolic Profiles

**Figure 6:**
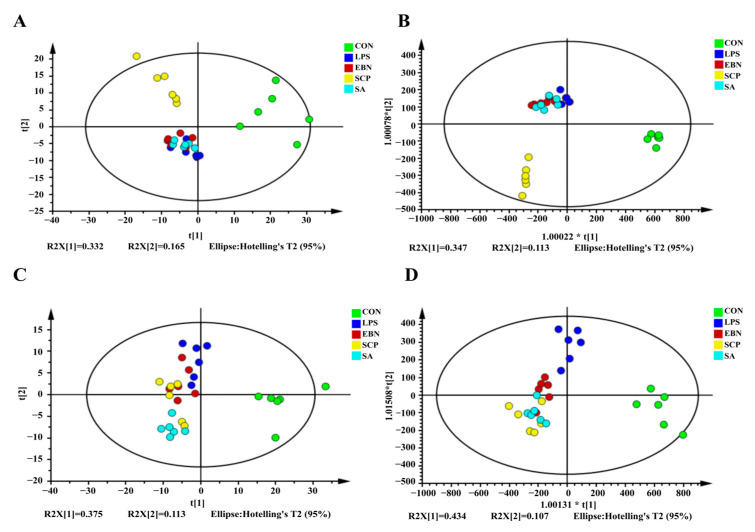
Pattern recognition analysis in positive and negative ion modes. (**A**,**C**) PCA diagram of positive ion mode and negative ion mode. (**B**,**D**) OPLS-DA diagram of positive ion mode and negative ion mode.

After preprocessing the data and imputing the missing values, 417 chromatographic peaks were detected in the positive ion mode, and 358 were found in the negative ion mode. To assess the variations in the metabolic profiles, principal component analysis (PCA) and orthogonal partial least squares-discriminant analysis (OPLS-DA) models were built. In the PCA score plots (Figure 6A,C), the metabolic profile of the LPS group showed a significant divergence along the first principal component (PC1, x-axis), which verified the successful establishment of the inflammation model 30. Along the second principal component (PC2, y-axis), the groups treated with EBN, SCP, and SA presented distinct metabolic changes compared to the LPS group. Significantly, the SCP group was clustered nearest to the CON group, suggesting its excellent ability to restore metabolic homeostasis. The OPLS-DA model further effectively amplified the differences among the groups (Figure 6B,D).

#### 3.5.2. Screening of Differential Metabolites

**Figure 7:**
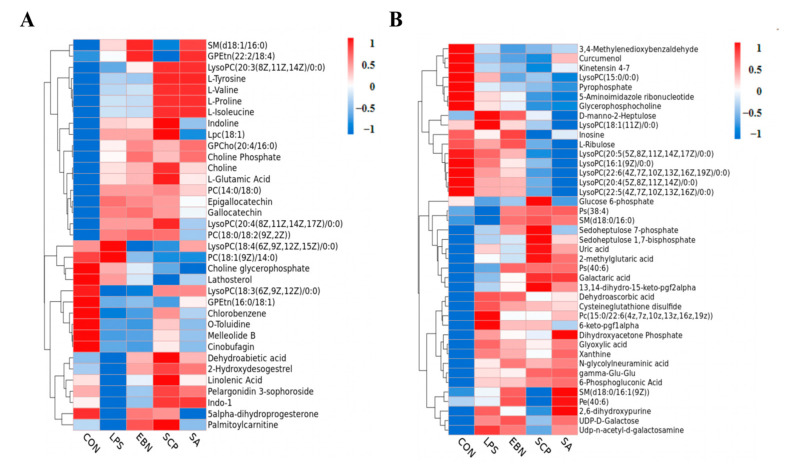
Clustering heat map of the expression of differential metabolites under positive and negative ion modes in the lung tissue of mice in each group. (**A**) Cluster heat map of differential metabolites in positive ion mode. (**B**) Cluster heat map of differential metabolites under negative ion mode.

In the metabolomics analysis, the screening of differential metabolites is usually based on the variable importance in the projection (VIP) values obtained from the OPLS-DA model. VIP describes the overall contribution of each variable to the model, as well as the *p* values obtained from the *t*-test. A threshold of *p* < 0.05 and VIP > 1 was set to screen out the differential metabolites with statistical significance. The differential metabolites among groups under the positive and negative ion modes were screened respectively (Figure 7). A total of 35 differential metabolites were identified under the positive ion mode, and 45 differential metabolites were identified under the negative ion mode. Compared with the CON group, a total of 39 metabolites in the LPS group showed significant changes in their contents (*p* < 0.05). Among them, the contents of 25 metabolites such as L-glutamic acid, xanthine, uric acid, and PC (14:0/18:0) in the LPS group increased significantly, while the contents of 14 metabolites such as glycerophosphocholine, pyrophosphate, o-toluidine, and GPEtn (16:0/18:1) decreased significantly. This indicated that LPS may cause metabolic disorders in the lung tissues of mice by interfering with these 39 metabolites. Compared with the LPS group, a total of 40 metabolites in the SCP intervention group showed significant changes in their contents (*p* < 0.05). Among them, the contents of 28 metabolites such as linolenic acid, choline, LPC (18:1), and indoline in the SCP intervention group increased significantly, while the contents of 12 metabolites such as glycerophosphocholine, xanthine, inosine, and LysoPC (18:1(11Z)/0:0) decreased significantly. This suggested that SCP may regulate the inflammation of the lung tissues in mice induced by LPS by interfering with these 40 metabolites.

#### 3.5.3. Metabolic Pathway Analysis of Differential Metabolites

**Figure 8:**
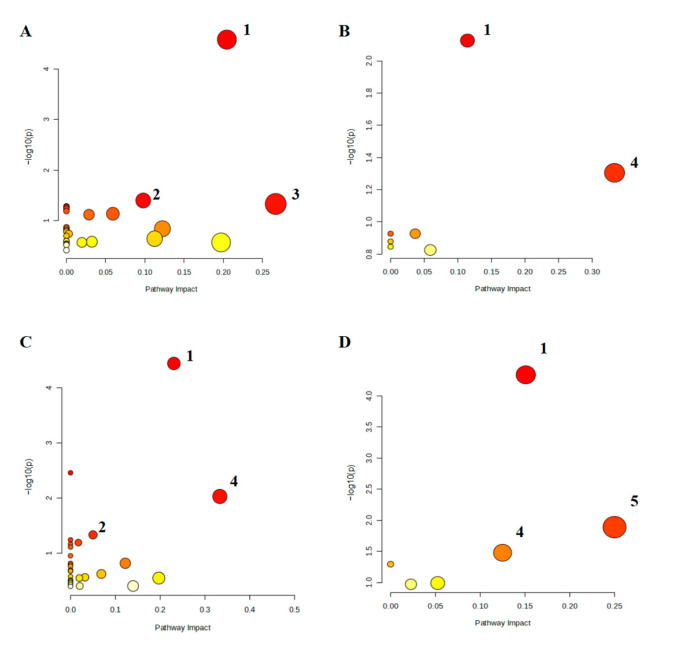
Heat map of differential metabolite expression in positive and negative ion modes in the lung tissue of mice in each group. (**A**–**D**) Differential metabolic pathways between the LPS and CON groups, the EBN and LPS groups, the SCP and LPS groups, and the SA and LPS groups. 1: glycerophospholipid metabolism, 2: purine metabolism, 3: glyoxylate and dicarboxylate metabolism, 4: alpha-linolenic acid metabolism, 5: linoleic acid metabolism.

Differential metabolites were mapped to the KEGG database to identify metabolic pathways with *p* < 0.05 and Impact > 0 (Figure 8). Compared to the CON group, the LPS group showed significant enrichment in glycerophospholipid metabolism, purine metabolism, glyoxylate, and dicarboxylate metabolism. Compared to the LPS group, the pathways enriched in EBN were glycerophospholipid metabolism and alpha-linolenic acid metabolism, the pathways enriched in SCP were glycerophospholipid metabolism, purine metabolism, and alpha-linolenic acid metabolism, and the pathways enriched in SA were glycerophospholipid metabolism, alpha-linolenic acid, and linoleic acid metabolism. Notably, the SCP group shared more overlapping pathways with the LPS group, suggesting its superior regulatory capacity at the metabolic pathway level.

## 4. Discussion

EBN proteins and carbohydrates are predominantly present as high-molecular-weight mucoproteins in the form of sialylated glycoproteins, which are characterized by low solubility and resistance to digestion [31]. In this study, EBN was digested using a two-step enzymatic hydrolysis protocol with Alcalase 2.4 L and papain. The contents of sialic acid (SA) and neutral sugars in SCP were both slightly higher than those in EBN, indicating that the optimization of the enzymatic hydrolysis process is conducive to the dissolution of neutral sugars and sialic acid [32]. Amino acids are the basic building blocks of proteins and polypeptides, and their composition and proportion are important factors influencing the physicochemical properties, biological activities, and other aspects of glycopeptides [33]. In this study, the contents of nine essential amino acids in SCP were abundant, according to the nutritional standards of essential amino acids recommended by the FAO/WHO. SCP has the potential to be an excellent choice for supplementing essential amino acids. Studies have shown that Asn is the main site where the sugar chain is linked to the protein during the *N*-glycosylation process, and Thr and Ser are the main sites where the sugar chain is linked to the protein during the O-glycosylation process [34]. The abundant contents of these three amino acids in SCP indicated that there may be certain glycosylation modifications in SCP.

It was revealed by electrophoretic analysis that glycoproteins retained high molecular weights due to the tight conjugation of glycan and protein. After hydrolysis, the molecular weights of glycoproteins were reduced, and non-glycan-conjugated proteins were further degraded into smaller peptides [22]. This observation suggested that peptide bonds in glycoproteins were selectively targeted by enzymatic cleavage; low-molecular-weight peptides were released while glycan chains covalently bound to residual peptide fragments were preserved. The resistance of glycan-peptide conjugated to enzymatic degradation implies that the cleavage sites of Alcalase 2.4 L and papain are likely to be distant from glycosylation sites in EBN glycoproteins. Thus, the integrity of the glycan was maintained during hydrolysis. Furthermore, SCP displayed typical peptide and carbohydrate features, with spectral profiles resembling those of quinoa and *Stichopus japonicus* glycopeptides [35,36]. Compared to EBN, enhanced 1401 cm^−1^ (−COO− symmetric stretching) and 1066 cm^−1^ (carbohydrate C-O) absorptions in SCP likely reflected increased SA and total carbohydrate content post-purification, confirming successful enrichment of glycan-rich moieties consistent with its sialylated glycopeptide identity.

The findings based on the C57BL/6 mouse model shared fundamental biological similarities with those based on human lung inflammation in LPS-induced mechanisms, and the experimental methods were universally used in the field, supporting the results’ reference value for similar inflammatory mechanism studies. LPS induced significantly elevated lung pro-inflammatory cytokine levels (TNF-α, IL-1β, and IL-6) and inflammatory cell counts (Neu, WBC, Eos, and Mon) in BALF, confirming the induction of lung inflammation. Increased inflammatory cell infiltration can reflect the immune system’s response to inflammatory stimuli, with cell counts as direct indicators of inflammatory severity [37,38]. In our study, all intervention groups significantly attenuated these inflammatory markers, with the SCP group demonstrating superior efficacy compared to EBN and SA groups, suggesting SCP as the primary bioactive component in EBN. In addition, the W/D ratio of lung issues, a key indicator of lung edema, was markedly increased in the LPS group, indicative of fluid accumulation in alveolar and interstitial spaces [39]. This observation was corroborated by histopathological analysis, which revealed alveolar structural damage, interstitial exudation, and neutrophil infiltration in the LPS group. Intervention groups alleviated lung tissue injury, with SCP exhibiting the most pronounced restorative effects. These findings align with prior studies showing that glycoprotein-bound SA exhibits enhanced anti-inflammatory activity compared to free SA [40].

Metabolomic profiling was conducted to elucidate SCP-mediated regulatory mechanisms on LPS-induced metabolic perturbations in the lung [41]. PCA and OPLS-DA demonstrated distinct metabolic profile alterations in the LPS group, with a clear separation between LPS and intervention groups, indicating metabolic homeostasis restoration. KEGG pathway enrichment analysis revealed that SCP primarily modulated glycerophospholipid, purine, and alpha-linolenic acid metabolism.

Glycerophospholipids, essential components of cellular membranes, are critically involved in membrane stability and functionality [42]. This study’s glycerophospholipid-related metabolites accounted for the highest proportion of differential metabolites. Moreover, choline and lysophosphatidylcholines (LysoPCs) have been identified as biomarkers associated with lipid metabolism-related chronic lung diseases [43]. Choline, a key precursor for phosphatidylcholine synthesis, is integral to membrane integrity. Under LPS-induced lung inflammation, elevated choline levels in the LPS group may reflect compensatory mechanisms to enhance choline synthesis or uptake for membrane repair [44]. Notably, SCP intervention increased choline content, suggesting that SCP may indirectly modulate choline metabolism by promoting synthesis or suppressing catabolism to support membrane homeostasis.

Purine metabolism dysregulation has been implicated in pulmonary injury and inflammation [45]. Xanthine, a key intermediate in purine metabolism, exacerbates oxidative stress and inflammatory responses when excessively accumulated. This study observed elevated xanthine levels in the LPS group, which may be attributable to the LPS-induced disruption of purine metabolic pathways, including enzymatic activation promoting xanthine synthesis [46]. Concurrently, LPS-mediated renal impairment increases uric acid production [47]. Following SCP intervention, xanthine levels were significantly reduced, suggesting the restoration of purine metabolism toward uric acid conversion. However, elevated uric acid levels persisted, potentially due to impaired renal clearance capacity. These findings indicate that SCP can alleviate LPS-induced lung inflammation partially through xanthine level modulation.

Alpha-linolenic acid, an essential fatty acid critical for maintaining physiological homeostasis, exhibits anti-inflammatory and antioxidant properties [48]. This study significantly reduced alpha-linolenic acid levels in the LPS group, likely due to its extensive consumption to counteract LPS-induced inflammatory responses [49]. Notably, alpha-linolenic acid levels were restored in the SCP intervention group. This restoration may be attributed to the SCP-mediated modulation of metabolic enzyme activity, which reduced inflammatory cell-driven alpha-linolenic acid depletion. These findings suggested that the anti-inflammatory effects of SCP against LPS-induced lung inflammation were mediated through the upregulation of endogenous alpha-linolenic acid levels.

## 5. Conclusions

In this study, SCP extracted from EBN exhibited typical structures of polypeptides and carbohydrates. EBN, SCP, and SA exerted effects on the level of pulmonary inflammation, the pathological structure of lung tissues, and the metabolic level of lung tissues, effectively improving the LPS-induced pulmonary inflammatory state in mice. Specifically, after SCP intervention, the number of Neu in BALF, the level of TNF-α in lung tissues, and the W/D ratio of lung tissues were significantly reduced. Moreover, the morphology of mouse lung tissues after SCP intervention was most similar to that of the CON group. SCP rectified the metabolic profiles of mice with pulmonary inflammation, regulated the expression of differential metabolites in lung tissues, such as choline, linolenic acid, and xanthine, and promoted the restoration of glycerophospholipid metabolism, alpha-linolenic acid metabolism, and purine metabolism pathways. However, the mechanism of action of SCP in vivo is complex and requires further investigation.

## Figures and Tables

**Table 1:** Amino acids distribution of SCP.

Amino Acids	Percentage Composition (%)	Amino Acids	Percentage Composition (%)
Asp	9.97 ± 0.04	Glu	8.25 ± 0.04
Cys	0.90 ± 0.02	Val *	8.03 ± 0.03
Ser	7.05 ± 0.02	Met *	1.07 ± 0.01
His *	3.56 ± 0.06	Phe *	6.89 ± 0.02
Gly	4.40 ± 0.00	Ile *	3.40 ± 0.01
Thr *	6.32 ± 0.02	Leu *	7.62 ± 0.01
Arg	7.14 ± 0.01	Lys *	4.13 ± 0.02
Ala	3.44 ± 0.01	Pro	10.39 ± 0.06
Tyr	6.23 ± 0.03	Trp *	1.22 ± 0.02

Aspartic acid (Asp), glutamic acid (Glu), cysteine (Cys), valine (Val), werine (Ser), methionine (Met), histidine (His), phenylalanine (Phe), glycine (Gly), isoleucine (Ile), threonine (Thr), leucine (Leu), arginine (Arg), lysine (Lys), alanine (Ala), proline (Pro), tyrosine (Tyr), tryptophan (Trp), * essential amino acids.

## Data Availability

The original contributions presented in the study are included in the article, further inquiries can be directed to the corresponding author.
